# Magnetic anchor technique-assisted thoracoscopic lobectomy in beagles

**DOI:** 10.1038/s41598-022-16050-4

**Published:** 2022-07-13

**Authors:** Yixing Li, Miaomiao Zhang, Aihua Shi, Peinan Liu, Hanzhi Zhang, Yong Zhang, Yi Lyu, Xiaopeng Yan

**Affiliations:** 1 Department of Hepatobiliary Surgery, The First Affiliated Hospital of Xi’an Jiaotong University, No. 277 West Yanta Road, Xi’an, Shaanxi 710061 People’s Republic of China; 2 Department of Thoracic Surgery, The First Affiliated Hospital of Xi’an Jiaotong University, Xi’an, Shaanxi People’s Republic of China; 3 National Local Joint Engineering Research Center for Precision Surgery & Regenerative Medicine, The First Affiliated Hospital of Xi’an Jiaotong University, Xi’an, Shaanxi People’s Republic of China; 4 Qide College, Xi’an Jiaotong University, Xi’an, Shaanxi People’s Republic of China

**Keywords:** Translational research, Surgery

## Abstract

In single-port thoracoscopic lobectomy, surgical instruments are likely to collide and interfere with each other. We used magnetic anchor technique to design an anchoring device suitable for thoracoscopic surgery, and verified the safety and feasibility of its use in animal experiments. Ten Beagles were used as models, and magnetic anchor technology was used to assist thoracoscopic lobectomy. During the operation, a self-designed magnetic anchored internal grasper was used in place of the traditional laparoscopic grasping forceps. The operation time, intraoperative blood loss, incidence of postoperative complications, and the effect of intraoperative use of the device were analyzed. All 10 beagles were successfully operated; the mean operation time was 19.7 ± 3.53 min (range 15–26 min), and the postoperative blood loss was < 10 mL. No surgical complications occurred. During the operation, the internal grasper was firmly clamped, the auxiliary operation field was well exposed, and the interference of the main operation hole instruments was effectively reduced. We provide preliminary experimental evidence of the safety and feasibility of magnetic anchor technique-assisted thoracoscopic lobectomy.

## Introduction

Lung cancer is the most common type of cancer in the world, and the main cause of cancer-related deaths. In 2020, an estimated 1.8 million people died of lung cancer across the world^1^. Thoracoscopic lobectomy is the most commonly used surgical method for the treatment of lung cancer. The traditional thoracoscopic operation requires three ports, which significantly reduces surgical trauma compared with thoracotomy. However, owing to the advances in the field of minimally-invasive surgery, fewer and smaller ports may further reduce postoperative pain^2^. In recent years, the concept of single operation hole and single-hole operation have been introduced in thoracoscopic surgery and applied in clinic^3^. However, after reducing the number of ports, all operating instruments need to enter and exit the thoracic cavity through the same operating hole, leading to collision and interference between instruments, forming a chopstick effect and increasing the difficulty of operation.

Magnetic anchor technique (MAT) entails the use of magnetic attraction between magnets and magnets, or magnets and paramagnetic substances, to make the anchoring magnets non-contact spatial anchoring of the target magnets^4^. MAT is currently widely used in laparoscopic surgery. It can facilitate cholecystectomy and appendectomy, but it has rarely been used in thoracoscopic surgery. Based on the MAT, our team designed a magnetic anchoring internal grasper suitable for thoracoscopic surgery to assist in the ports-reducing thoracoscopic surgery, and further verified its safety and feasibility in animal experiments.

## Materials and methods

### Ethical statement

The experiment was approved by the animal experiment ethics committee of the Xi'an Jiaotong University (ethics approval number: XJTULAC2019-1003). The research protocol and all experimental procedures were strictly in accordance with the Guidelines for the Care and Use of Experimental Animals issued by the Xi’an Jiaotong University Medical Center.

### Animals

Ten male Beagles (age: 1–6 years; weight: 8–12 kg) were used as the experimental group. This is an exploratory study, so there is no control group. The animals were obtained from the Experimental Animal Center, College of Medicine, Xi’an Jiaotong University (Xi’an, China) and acclimatized to laboratory conditions (23 °C, 12 h/12 h light/dark, 50% humidity, ad libitum access to food and water) for one week prior to commencing the experiments. All animal experiments are carried out in accordance with the Declaration of the Helsinki Convention on the Use and Care of Animals. Piperazine hydrochloride was injected intramuscularly every 12 h to relieve pain for two days after surgery, and 0.5 g cefazolin sodium was injected intramuscularly every 12 h to prevent infection for three days after surgery. At the end of the study, all animals were euthanized with excess barbital sodium (intravenous, 150 mg/kg pentobarbital sodium) and tissues were collected.

### Design of magnetic anchoring device

**Figure 1:**
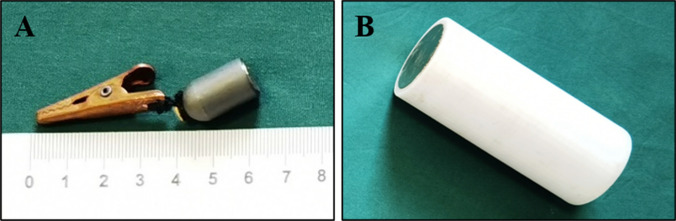
Magnetic anchoring device. (**A**) The internal grasper. (**B**) The anchoring magnet.

The magnetic anchoring device consists of an anchoring magnet and an internal grasper. The anchor magnet is a cylindrical magnet with a height of 140 mm and a diameter of 50 mm. The internal grasper is formed by connecting the target magnet and the tissue grasping forceps through a wire. The target magnet is a cylindrical magnet with a height of 15 mm and a diameter of 10 mm. The tissue grasping forceps is made of non-paramagnetic material. All magnets are made of N45 sintered neodymium iron boron material, and the surface is protected by nickel plating (Fig. 1).

### Determination of magnetic parameters

**Figure 2:**
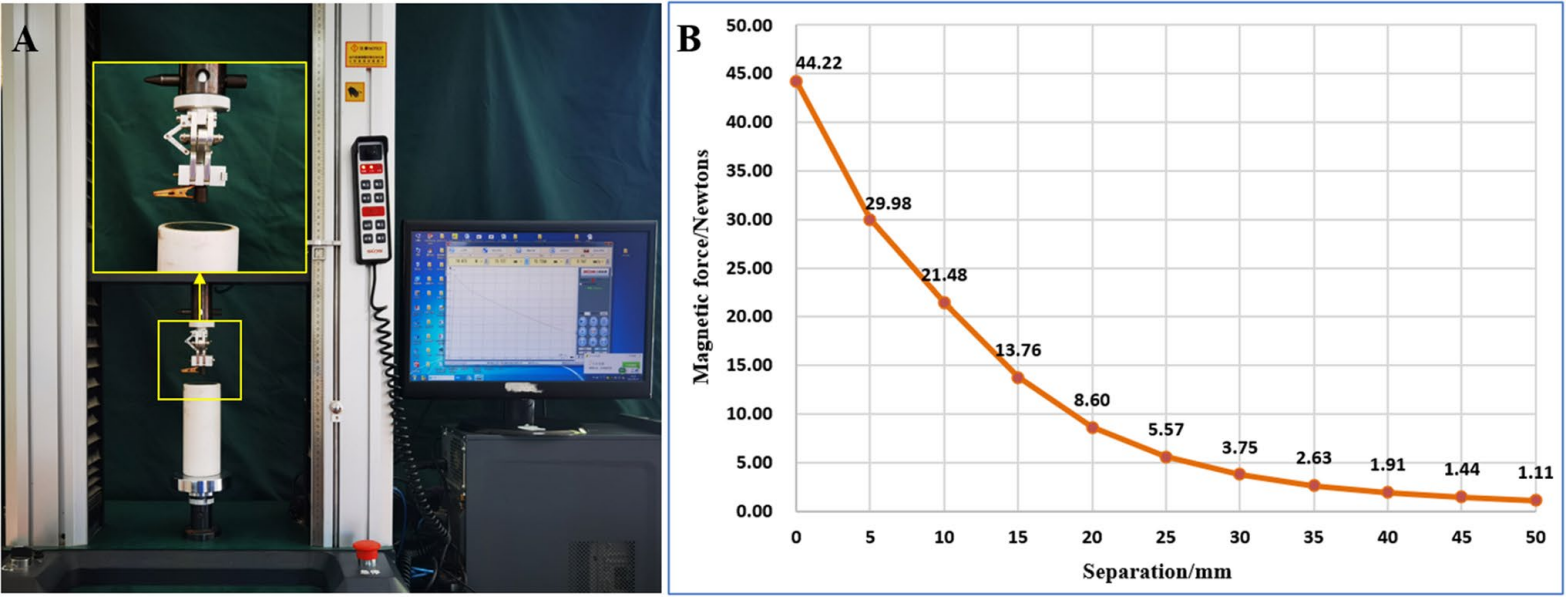
Measurement of magnetic force–displacement curve. (**A**) Schematic illustration of magnetic force–displacement curve measurement. (**B**) Magnetic force–displacement curve.

There is enough attractive force between the target magnet and the anchor magnet to play a traction effect. In line with the actual environment of thoracoscopy, we designed a cylindrical target magnet with a height of 15 mm and a diameter of 10 mm. We measured the change of its magnetic force with change in the distance between the two magnets (Fig. 2). We found that its traction can reach 44 N, and the existing dimensions can provide sufficient traction. In operation, the traction force can be flexibly adjusted by changing the position of the anchoring magnet according to the needs of tissue traction. The force of 2-8 N is sufficient for lobar traction in the Beagle.

### Surgical operation

**Figure 3:**
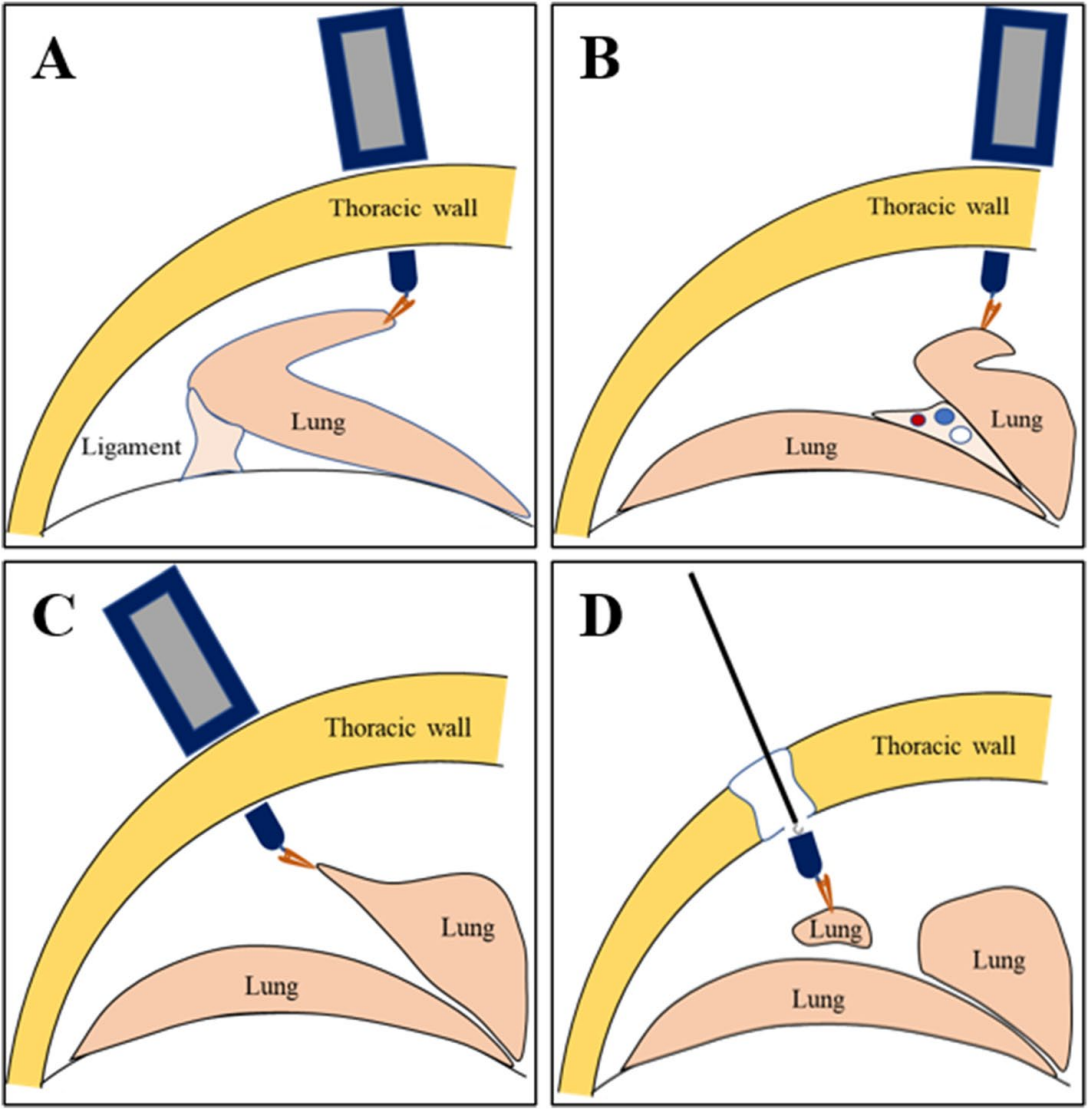
Schematic illustration of the use of a magnetic anchoring device in assisting thoracoscopic surgery. (**A**) Assist in exposing the hilum and separating the associated blood vessels. (**B**) Assist in the separation of ligaments. (**C**) Assist in the resection of lung tissue. (**D**) Take out the internal grasping forceps and lung tissue through the endoscopic device.

Before the operation, Beagles were fasted for 12 h and denied access to liquids for 6 h. Beagle dogs were anesthetized by intravenous injection of 3% pentobarbital sodium injection (1 mL/kg), and their left sides were fixed in a supine position. Intramuscular injection of cefazolin sodium 0.5 g was administered 30 min before surgery to prevent infection. After intubation of Beagle dogs with double-lumen endotracheal tube, the right lung was sealed and the left lung was ventilated. A small incision of approximately 1 cm in length was made in the 7th intercostal space in the right mid-axillary line, a trocar was inserted, and a thoracoscope was placed; a small incision of approximately 2 cm in length was made in the fourth intercostal space in the anterior axillary line as the main operation hole. We assumed a lesion and performed surgical resection. The internal grasper was inserted into the thoracic cavity from the main operation hole using titanium alloy forceps, and clamped around the target lung tissue. The position of the anchoring magnet was moved outside the body, and the target magnet was controlled by the magnetic field to pull the lung tissue and expose the surgical field (Fig. 3). The target magnet was used to pull the lung lobes to assist in the cauterization of lung ligaments and adhesion tissues. Subsequently, we changed the clamping position of the internal grasper to expose the interpulmonary fissures, and tried to dissect the hilum. We can also change the direction and strength of tissue traction by moving the anchor magnet throughout the procedure. After the blood vessels and trachea were separated and cleared, the internal grasper was clamped near the hypothetical lesion, and the position of the target magnet was changed to lift the lung lobe. Then a linear cutting stapler was used to excise the hypothetical lesion. After cutting the hypothetical lung lesion, we used the endoscopic instrument to attach to the tail of the internal grasper, and extracted it along with the target lung tissue through the main operation hole. The thoracic cavity was injected with water to expand the lungs, and no air leakage was observed at the cutting edge, and a thoracic drainage tube was inserted. We reserved sutures in a small incision in the mid-axillary line, and inserted a drainage tube to a suitable position in the chest cavity. The main operating hole was closed by stitching layer by layer. Subsequently, the anesthesiologist was asked to continue to expand the lungs. The drainage tube was placed under the surface of 500 mL bottled saline to expel the gas in the pleural cavity, and then the drainage tube was quickly pulled out and the incision was closed.

### Postoperative treatment

Intramuscular injection of pethidine hydrochloride was administered every 12 h for analgesia for two days after surgery, and intramuscular injection of cefazolin sodium 0.5 g was administered every 12 h for 3 days after surgery to prevent infection. We fed beagles regularly and monitored their condition. After 2 weeks of monitoring, all beagles were euthanized.

### Data collection and statistical analysis

Data pertaining to blood loss, operation time, postoperative complications, and other surgical parameters were collected. The intraoperative problems were also recorded. Descriptive analysis of the collected data was performed with SPSS 22.0 software. Normally distributed continuous variables were expressed as mean ± standard deviation while the categorical variables were expressed as frequency and percentage (%).

## Results

### Operation time, intraoperative blood loss, and the incidence of postoperative complications

All beagles successfully underwent thoracoscopic lobectomy assisted by the magnetic anchoring device. The average operation time was 19.7 ± 3.53 min (range 15–26 min). The intraoperative blood loss was < 10 mL. All beagle dogs showed good postoperative healing. There were no obvious complications such as pleural effusion, pneumothorax, severe wound infection, or blood in the lungs.

### The effect of intraoperative use of the device

**Figure 4:**
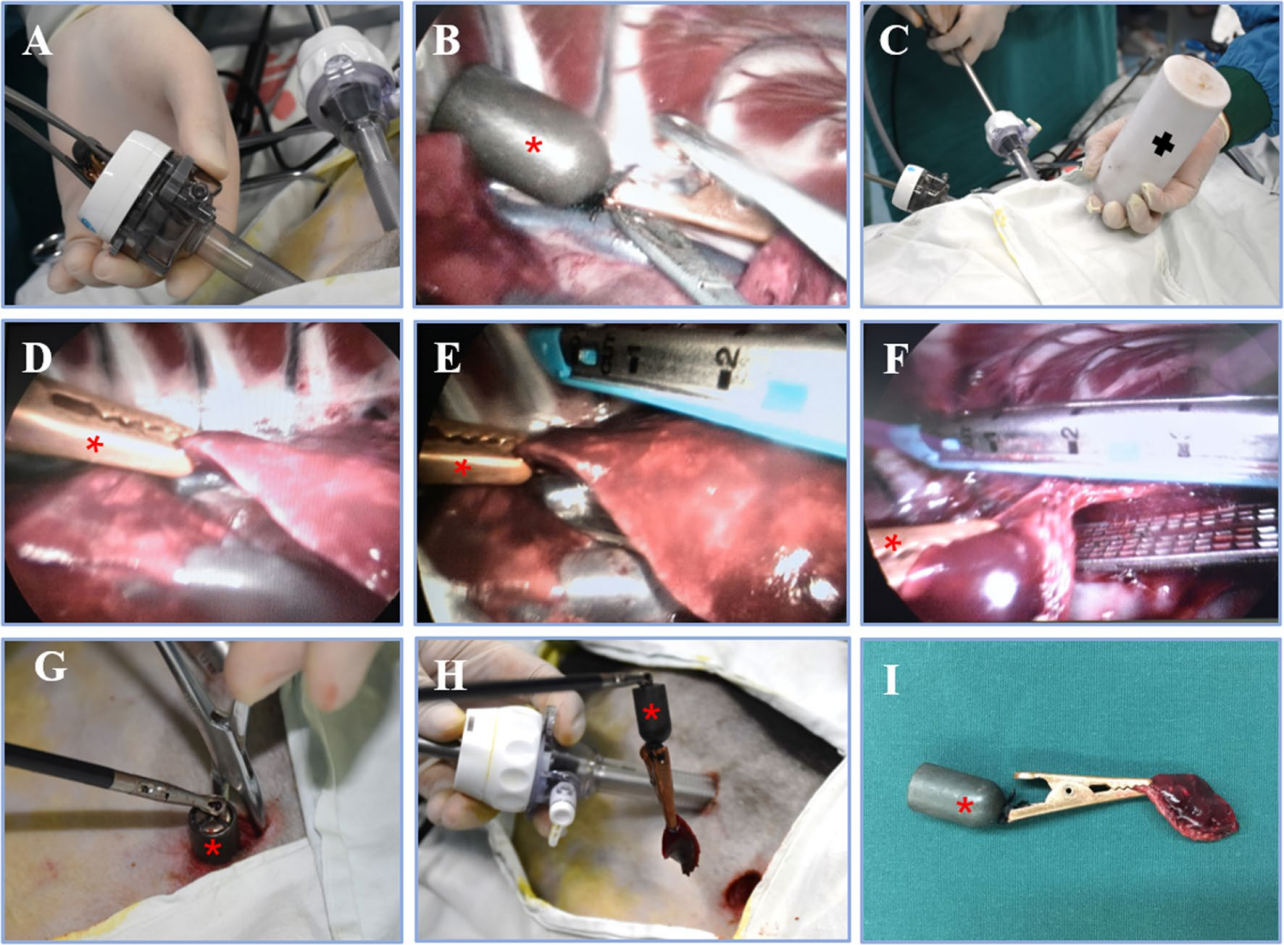
Intraoperative photographs showing the use of magnetic anchoring device to assist thoracoscopic surgery. (**A**) Use of titanium alloy forceps to insert the internal grasper into the body. (**B**) The internal grasper is clamped in a suitable position. (**C**) The position of the external anchoring magnet was moved to change the position of the internal grasper. (**D**) Assist in the exposure of lung fissures. (**E, F**) Assist in resection of lung tissue. (**G, H**) The internal grasper and target lung tissue is taken out. (**I**) The removed internal grasper and target lung tissue. (“*” shows the internal grasper, “ + ” shows the anchoring magnet.).

The magnetic anchor grasping forceps can clamp the lung lobes securely during the operation, without slippage, and successfully assisted the thoracoscopic surgery (Fig. 4). At the same time, the grasping forceps did not cause any tearing damage to the lung tissue. The external anchoring magnet can exert adequate traction force to pull the internal grasper for surgical operation. The use of a magnetic anchoring device can well assist the exposure of the surgical field. With its help, we successfully performed the resection of the hypothetical lesion. The magnetic anchoring device replaced the traditional endoscopic grasping forceps, effectively alleviating the collision and interference of equipment during the single-hole operation.

## Discussion

After thoracoscopic lobectomy was first proposed by Lewis in 1992^5^, the field of thoracoscopic surgery has shown rapid advances and has gradually replaced thoracotomy. Subsequently, thoracic surgeons pursued more minimally-invasive methods, and proposed single-port and double-port surgery. In 2004, Rocco G completed the first single-port thoracoscopic wedge resection^6^. Subsequently, efforts were made to further develop thoracoscopic surgery with reduced ports. Several studies have shown that reducing the number of ports reduces intraoperative bleeding, postoperative pain, drainage time, and hospital stay^3,7,8^. However, irrespective of the method, more operating instruments will inevitably need to pass in and out of the thoracic cavity through the same port, which is likely to cause instrument collision and interference with surgical operations^9,10^. In addition, it also increases the difficulty of learning for the surgeon^11^.

The magnetic anchor technique-assisted laparoscopic surgery was first reported by the Cadeddu team. They developed a magnetic anchoring and guidance system (MAGS) and proposed the replacement of some traditional laparoscopic instruments with this system^12^. They used this system to successfully perform transvaginal cholecystectomy^13^ and laparoscopic nephrectomy^14^ in several pigs. Clinical trials of this system were subsequently carried out^15^. The results demonstrated the safety and feasibility of magnetic anchor technique-assisted ports-reducing laparoscopic surgery, which can reduce instrument collisions and improve the operating space.

Kume et al. developed a magnet-retracting forceps^16^ and used it to pull out the gallbladder during laparoscopic cholecystectomy in a pig model. The device afforded good exposure of the surgical field. Subsequently, Dominguez et al. developed the TD-magnet system and used the device to perform cholecystectomy in 40 patients^17^. They solved the shortcomings of the device designed by Kume et al., separated the magnet and the gallbladder, and demonstrated a better range of motion. At the same time, Dominguez wrapped the magnets to allow simultaneous use of multiple magnets in the body.

Under the leadership of Professor Lv Yi, our team has summarized the application of magnetic force and further explored and conducted research in recent years. We proposed a magnetic surgical system, which incorporates a variety of technical theories. It mainly includes magnetic anchor technique (MAT), magnetic compression technique (MCT), magnetic navigation technique (MNT), magnetic levitation technique (MLT), magnetic tracer technique (MTT), and magnetic drive technique (MDT)^4^. Using the theory of magnetic anchor technology, we designed a magnetic anchoring internal grasper, which has been successfully applied to laparoscopic cholecystectomy^18^, laparoscopic hysterectomy^19^, and laparoscopic appendectomy.

At present, the use of magnetic anchor technology in thoracic surgery mainly includes the 3MP system for treatment of pectus excavatum^20^ and to assist thoracoscopic surgery. To the best of our knowledge, there are few reports on the use of magnetic anchor technology for thoracoscopic surgery. Martinez-Ferro et al. completed thoracoscopic lung wedge resection in a patient applying the magnetic anchoring device to single port thoracoscopic surgery in 2011^21^; however, they did not conduct further studies. We have accumulated experience in the application of magnetic anchored internal grasping forceps to assist laparoscopic puncture-reduction surgery in the early stage. Magnetic anchor technique is mostly used in laparoscopic surgery, but in fact, the bony structure of the chest wall provides more stable physical support and wider traction space than the abdominal wall. Therefore, we believe that magnetic anchor technique will have a better performance in thoracoscopic lobectomy. In this study, the magnetically anchored built-in grasping forceps were extended to assist thoracoscopic ports-reduction surgery, and we assessed the feasibility and safety of its use in beagle dogs. In assisted thoracoscopic puncture-reduction surgery, the magnetic anchored built-in grasping forceps provided good exposure of the surgical field. It allowed for good exposure and dissection of the blood vessels and bronchus. Moreover, the grasping forceps provided enough traction force to assist in cutting the lung tissue.

Compared with the TD-magnet system, the grasping forceps designed by us has a good range of motion and reduces the use of wires to control its activities. We use a silk thread to connect the internal grasper and the target magnet, so that the target magnet and the tissue grasper do not have to form a fixed angle, which increases the range of movement of the target magnet. The position of the anchoring magnet is adjusted to control the internal grasping forceps to expose the surgical field. At the same time, the distance between the anchoring magnet and the chest wall is adjusted to adjust the traction force. On the side of the target magnet, we adopt the magnetic shielding technology to effectively prevent the attraction between the target magnet and the cavity mirror device. At the same time, only the tail end of the target magnet is exposed, which is helpful in withdrawal of the target magnet. Although we have documented the variation in the magnetic force of the two magnets with change in the distance, it still requires the surgeon to learn by practice and gain experience in controlling the traction force during the operation; however, this does not entail a steep learning curve. The disadvantage is that it may necessitate an assistant to control the anchoring magnet, but if an effective external holding device is used, the need for assistance can be avoided. Sintered neodymium iron boron material is a commonly used material in magnetic anchor device at present^21–23^. The surface protection method can be nickel coating, titanium nitride coating and so on. This has been reported by other authors and in our previous articles. The material is safe for humans.

In this study, we only used a single target magnet to assist surgery. The MAGS developed by the Cadeddu team can replace multiple laparoscopic devices and accommodate multiple magnetic anchoring devices in the abdominal cavity. However, further studies are required to assess the feasibility of replacing the endoscopic grasping forceps with more magnetic anchoring devices in the thoracic cavity. We believe that magnetic anchoring devices should not be used too much. On the one hand, the capacity of the thoracic cavity is fixed, and its ability to accommodate multiple magnetic anchoring devices, like the abdominal cavity, needs to be further discussed. On the other hand, a magnetic anchor internal grasper is sufficient for thoracoscopic wedge resection, and the built-in target magnet may be interfered by multiple external anchoring magnets, thereby affecting the operation.

Although we have achieved experimental success, this research still has shortcomings. First of all, in our study, we mainly performed the resection of the right lung tissue, and did not make repeated attempts on multiple lung lobes. Further studies are required to assess the suitability of this device for other more complex disease models. Secondly, we did not make a statistical comparison of the effect of using or not using a magnetic anchoring device on the operation, but just made a judgment based on the surgeon’s experience. In addition, because there are still differences in the structure of beagles and humans, whether the device has the same effect in the human body needs further research. However, we believe that magnetic anchor technology can play a useful role in lung resection, lobectomy, and other operations. We can thoroughly extend this technology to single-port thoracoscopic surgery to achieve a more minimally invasive effect.

## Conclusion

The magnetic anchor grasping forceps designed based on magnetic anchor technique can assist in thoracoscopic surgery, effectively reduce the collision between instruments, and facilitate good surgical field exposure. We believe that the magnetic anchor technique has potential application value in thoracoscopic surgery and is worthy of further research.

## Data Availability

The datasets used and analyzed during the current study available from the corresponding author on reasonable request.

## References

[1] Sung H (2021). Global cancer statistics 2020: GLOBOCAN estimates of incidence and mortality worldwide for 36 cancers in 185 countries. CA Cancer J. Clin..

[2] Coletta D, Mascioli F, Balla A, Guerra F, Ossola P (2021). Minilaparoscopic cholecystectomy versus conventional laparoscopic cholecystectomy. J. Laparoendosc. Adv. Surg. Tech. A..

[3] Gonzalez-Rivas D (2013). Uniportal video-assisted thoracoscopic lobectomy: Two years of experience. Ann. Thorac. Surg..

[4] Yan XP, Shang P, Shi AH, Liu WY, Liu YX, Lv Y (2019). Exploration and establishment of magnetic surgery. Chin. Sci. Bull.-Chin..

[5] Lewis RJ, Caccavale RJ, Sisler GE, Mackenzie JW (1992). One hundred consecutive patients undergoing video-assisted thoracic operations. Ann. Thorac. Surg..

[6] Rocco G, Martin-Ucar A, Passera E (2004). Uniportal VATS wedge pulmonary resections. Ann. Thorac. Surg..

[7] Tu CC, Hsu PK (2016). Global development and current evidence of uniportal thoracoscopic surgery. J. Thorac. Dis..

[8] Abouarab AA, Rahouma M, Kamel M, Ghaly G, Mohamed A (2018). Single versus multi-incisional video-assisted thoracic surgery: A systematic review and meta-analysis. J. Laparoendosc. Adv. Surg. Tech. A..

[9] Wang L, Liu D, Lu J, Zhang S, Yang X (2017). The feasibility and advantage of uniportal video-assisted thoracoscopic surgery (VATS) in pulmonary lobectomy. BMC Cancer.

[10] Gonzalez-Rivas D, Yang Y, Ng C (2016). Advances in uniportal video-assisted thoracoscopic surgery: pushing the envelope. Thorac. Surg. Clin..

[11] Drevet G, Ugalde Figueroa P (2016). Uniportal video-assisted thoracoscopic surgery: safety, efficacy and learning curve during the first 250 cases in Quebec, Canada. Ann. Cardiothorac. Surg..

[12] Park S, Bergs RA, Eberhart R, Baker L, Fernandez R, Cadeddu JA (2007). Trocar-less instrumentation for laparoscopy: magnetic positioning of intra-abdominal camera and retractor. Ann. Surg..

[13] Scott DJ (2007). Completely transvaginal NOTES cholecystectomy using magnetically anchored instruments. Surg. Endosc..

[14] Zeltser IS, Bergs R, Fernandez R, Baker L, Eberhart R, Cadeddu JA (2007). Single trocar laparoscopic nephrectomy using magnetic anchoring and guidance system in the porcine model. J. Urol..

[15] Cadeddu J (2009). Novel magnetically guided intra-abdominal camera to facilitate laparoendoscopic single-site surgery: initial human experience. Surg. Endosc..

[16] Kume M (2008). A newly designed magnet-retracting forceps for laparoscopic cholecystectomy in a swine model. Minim. Invasive Ther. Allied Technol..

[17] Dominguez G, Durand L, De Rosa J, Danguise E, Arozamena C, Ferraina PA (2009). Retraction and triangulation with neodymium magnetic forceps for single-port laparoscopic cholecystectomy. Surg. Endosc..

[18] Bai JG (2019). Clinical application of magnetic anchor technique in laparoscopic cholecystectomy. J. Laparosc. Surg..

[19] Kang SR (2020). Experimental study on magnetic anchor device in assisting trocar-less laparoscopic hysterectomy. China Med. Devices..

[20] Graves CE (2017). Magnetic mini-mover procedure for pectus excavatum IV: FDA sponsored multicenter trial. J. Pediatr. Surg..

[21] Padilla BE, Dominguez G, Millan C, Martinez-Ferro M (2011). The use of magnets with single-site umbilical laparoscopic surgery. Semin. Pediatr. Surg..

[22] Shang Y (2017). An application research on a novel internal grasper platform and magnetic anchoring guide system (MAGS) in laparoscopic surgery. Surg. Endosc..

[23] Zhang L (2021). Internal grasper and magnetic anchoring guidance system in gynecologic laparoendoscopic single-site surgery: a case series. J. Minim. Invasive Gynecol..

